# Misunderstandings in ART Triadic Interactions: A Qualitative Comparison of First and Follow-Up Visits

**DOI:** 10.3389/fpsyg.2021.641998

**Published:** 2021-06-10

**Authors:** Maria Grazia Rossi, Elena Vegni, Julia Menichetti

**Affiliations:** ^1^Instituto de Filosofia da Nova, Faculdade de Ciências Sociais e Humanas, Universidade Nova de Lisboa, Lisbon, Portugal; ^2^Santi Paolo and Carlo Hospital, University of Milan, Milan, Italy; ^3^Institute of Clinical Medicine, University of Oslo, Oslo, Norway; ^4^Health Services Research Unit, Akershus University Hospital, Lørenskog, Norway

**Keywords:** misunderstanding, doctor–couple communication, assisted reproductive technology (ART), infertility care, shared understanding

## Abstract

**Background:**

Misunderstandings in medical interactions can compromise the quality of communication and affect self-management, especially in complex interactions like those in the assisted reproductive technology (ART) field. This study aimed to detect and describe misunderstandings in ART triadic visits. We compared first and follow-up visits for frequency, type, speakers, and topics leading to misunderstandings.

**Methods:**

We purposively sampled 20 triadic interactions from a corpus of 85 visits. We used a previously developed coding scheme to detect different types of misunderstandings (i.e., with strong, acceptable, and weak evidence). We analyzed also the different topics leading to strong misunderstandings (direct expressions of lack of understanding, pragmatic alternative understandings, semantic alternative understandings) to provide insights about the contents of the consultation that may need particular attention and care.

**Findings:**

We detected an overall number of 1078 misunderstandings in the 20 selected visits. First visits contained almost two-third of the misunderstandings (*n* = 680, 63%). First visits were particularly rich in misunderstandings with acceptable evidence (e.g., clarifications and checks for understanding), compared to follow-up visits. In first visits, doctors’ turns more frequently than couples’ turns contained misunderstandings, while in follow-up visits it was the other way around. Looking at the couple, the majority of the misunderstandings were expressed by the woman (*n* = 241, 22%) rather than by the man (*n* = 194, 18%). However, when weighting for their number of turns, 9% of the men’s turns included an expression of misunderstanding, compared to the 7% of the women’s turns. Finally, more than half of the misunderstandings with strong evidence were about history-taking and treatment-related topics, and while the history-taking ones were particularly frequent in first visits the treatment-related ones were more present in follow-up visits.

**Discussion:**

Findings indicate that first visits may deserve particular attention to avoid misunderstandings, as they are the moment where a shared understanding can be harder to reach. In particular, misunderstandings happening in first visits seem mostly related to physicians having to reconstruct the clinical history of patients, while those in the follow-up visits seem to reflect residual and unsolved doubts from the couple, especially concerning treatments.

## Introduction

Effective and efficient communication is paramount to improve patient trust and satisfaction with doctors (Chandra et al., 2018), patient safety and autonomy (Stewart, 1995; Street, 2013; Berger et al., 2017), patient adherence to treatment recommendations, and patients’ physical and mental health (Hall et al., 1988; Stewart, 1995; Zolnierek and Dimatteo, 2009). Poor communication may compromise information disclosure and higher malpractice claims (Levinson et al., 1997), increase patients’ dropout rates and doctor-shopping behaviors (Hagihara et al., 2005; Lynch et al., 2007), thus raising costs for healthcare systems. Communication has been also emphasized as the main tool for physicians to build their relationship with patients, fulfilling different functions in first and follow-up visits (Van Dulmen et al., 1997; Fossum and Arborelius, 2004; Graugaard et al., 2005). Building an affective connection and good relationship during the first visit has an important impact on follow-up visits, where less effort may be needed to maintain a positive and functional climate (Van Dulmen et al., 1997) and where patient evaluations may be particularly influenced by the affective connection with physicians established in first visits (Gulbrandsen et al., 2020). In particular, emotional and cognitive/informational aspects have been regarded to define mostly what counts as effective communication in the context of doctor–patient interactions (Di Blasi et al., 2001). This distinction has been used to emphasize the need to look after both contents and processes in clinical communication since what is communicated and how it is communicated are mutually interdependent factors (Cox and Li, 2020).

Communication is particularly challenging in the field of assisted reproductive technology (ART). A complex interlacement of medical, technical, and juridical language characterizes ART medical interactions. Besides, ART interactions are often triadic, with the physician handling the infertility problems of a couple. This implies dealing, most of the time, with two patients simultaneously, taking into account different or even contrasting socio-emotional and information needs in addition to diverse male/female infertility factors (e.g., to provide emotional support or to make informed decisions on semen collection, sperm washing vs. egg donation, fallopian tube examination). Previous research has focused primarily on the psychological effects of a diagnosis of infertility and ART care (Greil, 1997; Purewal et al., 2017; Samani et al., 2017; Stanhiser and Steiner, 2018; Courbiere et al., 2020). It is indeed well-known that fertility treatment is a source of stress both for couples and healthcare providers. From the couples’ side, the high levels of stress are due to the infertility issue itself and to the treatments that are both emotionally and physically demanding (Van den Broeck et al., 2010; Gameiro et al., 2012). Success rates are low, around 30% per cycle, with couples interrupting treatment or change the clinic due to patient dissatisfaction and distress (Van den Broeck et al., 2009; Gameiro et al., 2012). Because of the low success rates and because ART treatment is generally not fully covered by national insurance systems, doctors have to both endorse their services, manage their clients’ expectations, and, more often than not, deliver bad news, which means handling the psychological distress of patients (Leone et al., 2017). Gender differences in psychological reactions to infertility have been documented by various studies (Jordan and Revenson, 1999; Peterson et al., 2006; Nagórska et al., 2019), raising interesting questions on the role that male and female patients may play during ART visits. Evidence shows that male patients are less talkative during ART visits but also that lower male satisfaction is associated with the decision to change clinic (Borghi et al., 2019). Moreover, male partners are less likely to talk with other people about their experience of ART, which might explain why infertile men have high levels of psychological distress (Babore et al., 2017). All these aspects (uncertainty of outcomes, poor prognosis, socio-emotional and communication complexity, gender differences) might increase the risk for misunderstandings in ART triadic interactions. However, doctor-patient communication has been still poorly investigated in the field of ART (but see Rossi et al., 2017; Leone et al., 2018; Borghi et al., 2019), and there have been no studies looking at how understanding is interactionally achieved or detained in real-life ART consultations.

Shared or mutual understanding is a defining feature of effective communication. As a process of negotiation and co-construction of meanings, reaching shared understanding is indeed important in all phases of medical consultation and is one of the key communication goals in the medical context (Rossi and Macagno, 2020). Problems of understanding might lead to wrong or delayed diagnosis and suboptimal adherence to treatments (Street et al., 2009; Epstein and Street, 2011). To achieve a shared understanding, communication needs to be adjusted to each patient’s individual needs and also to take into account the specific clinical tasks at different care stages. As mentioned before, first visits usually fulfill different functions from follow-up visits: they are longer, physicians may undertake extensive and quite complex segments of history-taking (Fossum and Arborelius, 2004), and tend to adopt a more task-focused communication style (Graugaard et al., 2005). We can therefore expect a major difficulty in obtaining shared understanding in first visits, with the result of increasing misunderstandings’ number and dangerousness. However, while misunderstandings have been deeply investigated in other communicative settings, and especially in multilingual and intercultural contexts (Angelelli, 2004; Roberts and Sarangi, 2005; Roberts et al., 2005; Schouten and Meeuwesen, 2006; Paternotte et al., 2015; Cox, 2017; Crawford et al., 2017; Cox and Li, 2020; Rossi and Macagno, 2021), only a few studies have systematically analyzed problems of understanding in healthcare settings (McCabe and Healey, 2018). Even beyond the ART context, to the best of our knowledge, no study compared difficulties in reaching a shared understanding between first and follow-up visits.

This study aimed to describe and compare problematic understandings in first and follow-up ART triadic visits. In particular, the study aimed to: (1) report how many misunderstandings were expressed in first and follow-up visits, of which type, and by whom; (2) identify topics leading to strong misunderstandings.

## Materials and Methods

### Source Material

We purposively selected 20 videotaped interactions between 10 doctors and 20 couples from a corpus of 85 videos collected in eight private and public ART Italian clinics between 2013 and 2015. This subsample of interactions was selected based on the following criteria: (1) to contain triadic consultations only, and (2) to pair first and follow-up consultations performed by the same doctors. The latter criterion was chosen to make a comparison between first and follow-up visits possible and not biased by doctors’ characteristics and communication styles. The demographic and clinical characteristics of the participants are shown in Table 1. Couples had unprotected sex for a mean of 3.2 years before the consultation (range, 1–9). Mixed and idiopathic were the most frequent causes of infertility in this group of patients. The second-level intervention (IVF/ICSI) was offered to 50% of participants, with a favorable prognosis in 60% of the cases.

**TABLE 1:** Participant sociodemographic and clinical characteristics.

Patient characteristics	Value
**Participant age, mean years (SD), range**	
Females	38.95 (4.1), 33–49
Males	41.75 (7.35), 32–64
Unprotected sex, mean years (SD), range	3.2 (2.6), 1–9
**Cause of infertility, n (%)**	
Female factor	6 (30)
Male factor	1 (5)
Other factors	13 (65)
Mixed	6 (30)
Idiopathic	6 (30)
Not evaluable	1 (5)
**Therapeutic indications, n (%)**	
IUI	1 (5)
IVF/ICSI	10 (50)
Not recommended	2 (10)
Waiting	3 (15)
Heterologous (use of donor gametes)	3 (15)
**Prognosis, n (%)**	
Favorable	12 (60)
Unfavorable	7 (35)
Unknown	1 (5)
**Physician characteristics Gender, n (%)**	
Female	8 (80)
Male	2 (20)
**Participant age, mean years (SD), range**	
Female	46.1 (9.3), 34–62
Male	51.5 (11), 42–61
**Participant years in practice, mean years (SD), range**	
Females	16.75 (10), 3–33
Males	16 (4.6), 12–20

The corpus was video recorded and collected with the informed consent of all participants, who gave their consent to use their video for communication studies. The research project was approved by the Ethical Review Board of the University of Milan and by the Ethical Review Boards of the eight participating ART clinics. The subsample of data was subsequently transcribed, following a simplified version of Jefferson’s transcription system (Jefferson, 2004).

### Data Analysis

#### Analysis of Problematic Understanding

Different types of misunderstanding were systematically analyzed by adopting an already existing coding scheme grounded on an interactional view of communication (Rossi and Macagno, 2020). The coding scheme was developed to embrace a wide range of understanding failures, thus including not only strong misunderstandings but also potential misunderstandings with weak linguistic evidence, like irrelevant turns or lack of uptakes (see also Tzanne, 2000; Rossi and Macagno, 2020). Therefore, considering key distinctions made in pragmatics and linguistics (Bazzanella and Damiano, 1999a,b; Weigand, 1999; Yus, 1999; Verdonik, 2010), it included different types of problematic understandings, grouped into three main categories based on their strength of linguistic evidence.

Following the procedures described in previous studies (Macagno and Rossi, 2019; Rossi and Macagno, 2020), two researchers (MGR and JM) independently worked on the transcripts of the consultations and detected the seven different types of problematic understandings considered by the coding scheme. The two researchers met several times along the process to discuss doubts, and a third researcher (EV) was involved in case of disagreement. The final sample of identified misunderstandings was then analyzed by grouping the seven types of problematic understandings in three main pre-defined and mutually exclusive categories, based on the misunderstandings’ linguistic evidence: strong (lack of understanding, semantic alternative understanding, and pragmatic alternative understanding), acceptable (clarification and check for understanding), and weak (no uptake and irrelevance) evidence. The “strong evidence” category thus includes actual misunderstandings, while the “acceptable evidence” category captures cues of doubtful understandings. Finally, the “weak evidence” category captures indirect signs of potential misunderstandings, as a lack of coherence between interlocutors’ turns. Table 2 offers a brief description and an example for each coding category.

**TABLE 2:** Coding categories (name, description, example).

Main category and sub-categories	Description	Example
Strong evidence		
*Lack of understanding (LACK)*	The hearer acknowledges explicitly that s/he cannot understand, or that the interpretation that s/he has achieved is not acceptable	FIRST-VISIT
		Infertility cause: “unknown”; treatment: “waiting.”
		D: So this is the only test that I suggest you do here or in “X” [name of another health care facility]
		*Questo ecco è l’unico esame che le consiglio di fare qui::(.) o in alternativa a “X” [nome di un’altra struttura sanitaria]*
		MP: uh
		*ah*
		D: not elsewhere because it’s a test one of few tests that is still done manually
		*diciamo non da altre parti perché è un esame è uno dei pochissimi esami che ancora viene eseguito manualmente*
		MP: uh huh
		*mh mh*
		D: so the lab technician that looks at it and their experience is important
		*quindi l’operatore che lo vede e la sua esperienza è fondamentale*
		MP: uh huh
		*mh mh*
		D: since it’s not a simple, pleasant test
		*dato che non è un esamino simpaticissimo*
		**MP: um I don’t think I… what do you mean manually? *cioè non ho capito manualmente cosa vuol dire?***
*Semantic alternative understanding (SEM ALT)*	The hearer interprets the speaker’s turn by specifying its meaning in a way that is not acceptable or accepted, and the speaker corrects this alternative interpretation. The interpretation is about the semantic representation of an utterance.	FIRST-VISIT
		Infertility cause: “female infertility”; prognosis: “unfavorable.”
		FP: the doctor gave me these, they told I have to do preventive treatment
		*il dottore mi ha dato questi: mi ha detto che devo fare la [profilassi]*
		D: yes then you should take them
		*[si] li deve prendere, allora*
		FP: I should take them
		*Li devo prendere*
		D: yes, then you should take them
		*sí sí li deve prendere (.) allora (unint)*
		FP: I went to the bathroom, I saw blood it’s norma-
		*sono andata in bagno ho visto sangue è norma-*
		D: that’s normal, that’s normal “FP surname” allright (.) “FP name”
		*è normale è normale (.) “cognome di FP” va bene (.) “nome di FP”*
		FP: yeah
		*si::*
		D: great, and so everything is fine
		*Benissimo (2.0) e quindi questo siamo apposto*
		(10.0)
		**FP: do you say, um, that’s totally normal?** *quindi per lei cioè mh voglio sape- è norma- cioè è tutto: [non]*
		D: [no] listen ma’am it’s not normal in the sense that having an FSH a little high being over 40, that happens, you have to see if it stays that way. Plus the main issue is that it plays against you a bit for your fertility
		*no ascolti signora non è normale nel senso (.) fsh un pochino elevato (.) dopo i 40 anni succede bisogna vedere se è una cosa fissa e poi soprattutto il problema è (.) che le gioca un pochino contro per la fertilità*
*Pragmatic alternative understanding (PRAG ALT)*	The hearer interprets the speaker’s turn by drawing inferences that are not acceptable or accepted, and the speaker corrects this alternative interpretation. The interpretations is about the intended purpose of a speaker’s utterance.	FOLLOW-UP
		Infertility cause: “mixed”; treatment: “second-level”; prognosis: “favorable”
		FP: uh the last question, from the day of the: sample til the transfer day is it better to take a few days off and stay home?
		*eh come ultima domanda dal giorno del: prelievo al giorno del transfert è meglio avere qualche giorno a casa?*
		**D: we can give you it [medical leave], if you wish, yes**
		*glielo diamo, se lo desidera sì*
		FP: no, I’m asking what’s best
		*no io chiedo quello che è meglio*
**Acceptable evidence**		
*Clarification (CLA)*	The hearer asks the speaker to specify the meaning of an utterance, as it can have different interpretations. No interpretative hypothesis is advanced; only a question is asked to disambiguate a speaker’s utterance (or one of its components).	FOLLOW-UP
		Infertility cause: “idiopathic”; treatment: “second-level”; prognosis: “unfavorable”
		MP: so there’s another thing I needed- uh. after the transfer
		*ma c’è un’altra cosa che dovevo- ah. dopo il transfer*
		D: yes
		*sí*
		MP: she can walk no problem?
		*lei può: camminare [tranquillamente:?]*
		D: not a problem
		*[tranquillamente]* =
		MP: ok
		*[sì]*
		D: her daily life, we don’t recommend rest
		= *una vita normale, noi non consigliamo riposo.*
		MP: so uh
		*cioè [mh:]*
		**FP: I mean because I drive, I mean can I go back to school without a problem**
		*[cioè] perchè io guido, cioè posso tornare a scuola [tranquillamente]*
		D: not a problem, of course
		*[tranquillamente, certo]*
*Check for understanding (CHECK)*	The hearer expresses a doubt of understanding, as s/he is uncertain to have understood correctly what the speaker said.	FIRST-VISIT
		Infertility cause: “idiopathic”; treatment: “second-level”; prognosis: “favorable”
		FP: really the appendix was fantastic and when they opened me up they said who decided this? So they decided to do it on me and when they obviously decided to take it out, they found a cyst on the right ovary
		*in realtà l’appendice era fantastica e quando mi hanno aperto hanno detto ma chi è quello che l’ha decisa? Che poi aveva decisa di farmela: e quando l’hanno ovviamente tolta () deciso di toglierla, hanno trovato una ciste sull’ovaia destra*
		**D: the right one**
		*destra*
		FP: yes
		*sì*
		**D: so your pain wasn’t probably from your appendix**
		*(0.3) per cui lei aveva dolore probabilmente non per [l’appendice]*
		FP: exactly
		*[brava]*
		**D: but from the cyst** *ma per la ciste*
		FP: exactly
		*esatto*
**Weak evidence**		
*Irrelevance (IRR)*	The hearer continues the conversation with a turn that is incoherent either pragmatically (e.g., request of information followed by an acknowledgment) or for topic (change of subject) with the previous turn.	FIRST-VISIT
		Infertility cause: “female infertility”; treatment: “heterologous”; prognosis: “favorable”
		FP: because we found out that now in Italy the law has passed
		*perché abbiamo saputo che adesso in Italia: è stata consentita la legge* =
		D: you found out from the newspapers or
		= *l’avete sentito cosi sui giornali su*
		FP: yeah, from the newspapers and we wanted to know more about what you all do and where it’s at
		*si sui giornali e volevamo capire anche ↑che cosa facevate voi e su che punto era:*
		**D: sure sure but listen why don’t you tell me about your story?**
		*((nods)) certo certo ascoltate invece [mi raccontate pero la storia vostra?]*
*no uptake (NO UP)*	The hearer fails to take into account the other’s turn by interrupting the dialogue (silence) or continuing the dialogue without considering the interlocutor’s turn.	FIRST-VISIT
		Infertility cause: “mixed”; treatment: “second-level”; prognosis: “favorable”
		D: the second thing is the lesion caused by the needle Because you enter the belly with a needle, you know how IVF works
		*seconda cosa lesione da ago no? perchè si entra con ago nella pancia, lei sa come funziona la fiveat?*
		FP: yeah, a little, a little bit. But I would like you to explain it a little better, to him too, so that way we’re
		*un pochin sì un pochino si però vorrei che lo spiegasse un pò bene anche a lui perchè così siamo:-*
		D: ok
		*okey*
		FP: we have no risk of misunderstanding *nonabbiamo rischi di fraintendimento*
		**D: great um ((writes in folder)) married since?**
		*perfetto ehm: ((scrive cartella)) sposati dal?*
		FP: uh officially since 2011
		*ehm:ufficialmente dal [[2011]]*

*Evidence of problematic understanding is in bold. D, doctor; FP, female patient; MP, male patient. For all the details on the coding procedure see Supplementary Material annexed in Rossi and Macagno (2020).*

Videos of the consultations were stored in an encrypted hard disk at the University of Milan, and only the anonymized transcripts were used for the analysis. Types of problematic understandings were detected using Microsoft^®^ Office Excel (Office 365) and reported by using descriptive statistics (frequency; average; percentage). Inter-rater reliability (IRR) was conducted on 12 interviews (60%) which were independently analyzed by two researchers (MGR and JM); one researcher (MGR) concluded the analysis on the remaining 8 visits. IRR was strong (agreement 98%, Cohen’s Kappa > 0.80), except for the weak evidence for which Cohen’s Kappa value was only 0.5 (26 disagreements; 5001 agreements) (McHugh, 2012).

#### Analysis of Topics

Strong misunderstandings were also further analyzed to detect the main connected topics. This analysis followed an inductive process and the principles of thematic analysis (Braun and Clarke, 2006). In particular, we detected the explicit contents of misunderstanding within the data, meaning the direct object of e.g., a lack of understanding or other types of strong misunderstandings. As a first step, the content of each misunderstanding was extracted using the exact words adopted by the speaker. This resulted in a list of word-by-word contents. Then, as a second step, more generic and brief codes were tagged to the items. The next step was the generation of themes based on the similarities/differences between codes. The last step was completing the allocation of codes in the emerging themes for all the items, and checking the entire analytical process. Therefore, the analysis was an iterative process of refinement of codes and themes, where first codes and themes were generated, checked within the entire dataset, revised, and finally applied to the sample of ART visits. This process was performed by one researcher (MGR) in a constant discussion of cases with a second researcher (JM). Emerging themes were discussed with a third researcher (EV) and doubts were solved through discussion. Descriptive statistics (frequency; average; percentage) were used also in this case to report data on the emerging themes.

## Results

Overall, we found 1078 (11%) turns with misunderstandings over a total of 9941 turns in the 20 analyzed consultations. On average, there were 54 misunderstandings per visit (median = 43; range = 18–145).

### Type of Misunderstandings: Comparing First and Follow-Up Consultations

Most of the misunderstandings retrieved in the corpus were check for understandings (*n* = 641; 59%) and clarifications (*n* = 250; 23%) within the category of “acceptable evidence” (*n* = 891; 83%). Then, we retrieved 150 (14%) misunderstandings with a “strong evidence,” and in particular pragmatic alternative understandings (*n* = 62; 6%), semantic alternative understandings (*n* = 59; 5%), and lack of understandings (*n* = 29; 3%). The least represented types of misunderstanding were no uptakes (*n* = 20; 2%) and irrelevance (*n* = 17; 2%), in the “weak evidence” category.

The 63% (*n* = 680, the 13% of the first visits’ turns) of the misunderstandings occurred in the first visits compared to the 37% (*n* = 398, the 8% of the follow-ups’ turns) retrieved in the follow-ups. When comparing first and follow-up consultations for the different types of misunderstandings, we found that misunderstandings with acceptable evidence (clarification and check for understanding) mostly occurred in the first visits (65% vs. 35% of follow-ups), while those with weak evidence (and in particular irrelevant turns) mostly happened during follow-up consultations (54% vs. 46% of first visits). Differences between first and follow-up visits for the “strong evidence” category were mixed: pragmatic alternative understandings tended to occur slightly more frequently in follow-ups than first visits, while semantic alternative understandings usually occurred during first visits. Table 3 shows the frequency of the different types of misunderstandings in first visits and follow-ups.

**TABLE 3:** Frequency (n, %) of the different types of misunderstanding in first and follow-up visits.

Types of misunderstanding	First visits	Follow-up visits	Tot
**Strong evidence**	**85 (57%)**	**65 (43%)**	**150 (14%)**
*Lack of understanding*	*18*	*11*	*29*
*Semantic alternative understanding*	*38*	*21*	*59*
*Pragmatic alternative understanding*	*29*	*33*	*62*
**Acceptable evidence**	**578 (65%)**	**313 (35%)**	**891 (83%)**
*Clarification*	*160*	*90*	*250*
*Check for understanding*	*418*	*223*	*641*
**Weak evidence**	**17 (46%)**	**20 (54%)**	**37 (3%)**
*Irrelevance*	*6*	*11*	*17*
*No uptake*	*11*	*9*	*20*
**Tot misunderstandings**	**680 (63%)**	**398 (37%)**	**1078 (100%)**
**Turns tot**	**5212 (52%)**	**4729 (48%)**	**9941 (100%)**
**Misunderstandings/turns**	**13%**	**8%**	**11%**

### Misunderstandings in First and Follow-Up Consultations

When looking at who (doctor, female patient, male patient) expressed the misunderstanding, we found that misunderstandings were more frequently expressed by doctors (in doctors’ turns) (*n* = 643/1078, 60%) rather than by couples (*n* = 435/1078, 40%). Within the couple, the majority of the misunderstandings were expressed in women’s turns (*n* = 241/1078; 22%) rather than in men’s turns (*n* = 194/1078, 18%). However, when weighting for the number of turns, 9% of the men’s turns included an expression of a misunderstanding, compared to the 7% of the women’s turns.

The distribution of misunderstandings between doctors and couples differed between first visits and follow-ups: if in the first visits the doctors contributed to more than the two-third of the misunderstandings (*n* = 469/680, 69%), in the follow-ups doctors’ contributions went down to less than the half (*n* = 174/398, 44%). The reasons were clear when observing the types of misunderstandings: doctors expressed the majority of the requests for clarification and checks for understandings in the first visits (*n* = 422). For the other categories of misunderstandings, doctors and patients contributed more or less equally. Couples more frequently than doctors expressed strong types of misunderstandings both in first and follow-up visits, but there were no differences in the types of misunderstandings between female and male patients. Table 4 shows the frequencies of the different types of misunderstandings for first visits and follow-ups, distinguishing between doctors and couples.

**TABLE 4:** Types of misunderstanding in first and follow-up visits (*n* = 1078).

	First visits		Follow-ups	
Types of evidence	Doctors	Couples	Doctors	Couples
**Strong evidence**	**39 (26%)**	**46 (31%)**	**26 (17%)**	**39 (26%)**
*Lack of understanding*	8	10	5	6
*Semantic alternative understanding*	19	19	9	12
*Pragmatic alternative understanding*	12	17	12	21
**Acceptable evidence**	**422 (47%)**	**156 (18%)**	**138 (15%)**	**175 (20%)**
*Clarification*	116	44	34	56
*Check for understanding*	306	112	104	119
**Weak evidence**	**8 (22%)**	**9 (24%)**	**10 (27%)**	**10 (27%)**
*Irrelevance*	4	2	1	8
*No uptake*	4	7	9	2
**Total**	**469**	**211**	**174**	**224**

### Challenging Topics in First and Follow-Up ART Visits

We detected six main areas of topics connected to the strong misunderstandings: (a) history-taking topics (*n* = 51/150; 34%); (b) treatment-related topics, meaning the timing and procedures of treatments (*n* = 32/150: 22%); (c) clinical consultation topics, meaning the medical information exchanged during the consultation (*n* = 24/150; 16%); (d) bureaucratic topics, meaning the country and center rules (*n* = 22/150; 15%); (e) emotional topics, meaning concerns and complaints (*n* = 14/150; 9%); (f) relationship-building topics (*n* = 6/150; 4%). Details about the categories and subcategories of topics are reported in Appendix 1. Strong misunderstandings about history-taking topics were more frequent in first visits (*n* = 39) than in follow-ups (*n* = 12), while those about treatments were slightly more frequent in follow-ups (*n* = 19) than first visits (*n* = 13). For the other topics, misunderstandings were detected more or less in the same amount in first visits and follow-ups. Table 5 provides information about the topics and subtopics connected to the strong types of misunderstandings in first and follow-up visits.

**TABLE 5:** Topics connected to the strong types of misunderstanding in first and follow-up visits (*n* = 150).

Topics and sub-topics	First-visits	Follow-ups	Tot
*(A) Bureaucratic topics: Country and center rules*	10 (45%)	12 (55%)	22 (15%)
*(B) Treatment topics: Timing and procedures*	13 (41%)	19 (59%)	32 (22%)
*(C) Clinical consultation topics: Medical information*	12 (50%)	12 (50%)	24 (16%)
*(D) History-taking topics*	39 (76%)	12 (24%)	51 (34%)
*(E) Emotional topics: Concerns and complaints*	8 (57%)	6 (43%)	14 (9%)
*(F) Rapport-building topics*	3 (50%)	3 (50%)	6 (4%)
**Total**	**85 (57%)**	**65 (43%)**	**150 (100%)**
*Number of turns*	*5212 (52%)*	*4729 (48%)*	*9941 (100%)*
*Misunderstandings/number of turns*	*2%*	*1%*	*2%*

*(See Appendix 1 for more details on the sub-categories used).*

Strong misunderstandings about history-taking topics were more frequently expressed by doctors (*n* = 32/51) than by patients (*n* = 19/51), as were those about emotional topics (*n* = 5/6 in doctors’ turns) (Table 6). On the opposite, strong misunderstandings about bureaucratic, treatment-related, and clinical consultation topics were more frequently found in patients’ turns (*n* = 17/22, *n* = 25/32, *n* = 16/24, respectively) than doctors’ turns. While female patients expressed most of those about treatment-related and clinical consultation topics (*n* = 16/25 and *n* = 10/16, respectively), male patients most frequently expressed those about bureaucratic topics (*n* = 10/17).

**TABLE 6:** Topics connected to the strong types of misunderstandings (*n* = 150).

	Doctors	Couples
*(A) Bureaucratic topics: Country and center rules (n* = *22)*	5	17
*(B) Treatment topics: Timing and procedures (n* = *32)*	7	25
*(C) Clinical consultation topics: Medical information (n* = *24)*	8	16
*(D) History-taking topics (n* = *51)*	32	19
*(E) Emotional topics: Concerns and complaints (n* = *15)*	8	7
*(F) Rapport-building topics (n* = *6)*	5	1
	**65**	**85**

### Unpacking the Technical, Informational, and Emotional Complexity

We detected various examples showing the complex interlacement between technical information exchange and emotionally charged experiences that feature the ART field and which can particularly generate misunderstandings. As an example of this, we present an extract of an analyzed consultation.

In this consultation, the doctor advised the male patient to perform the semen analysis. Being a “manual” examination, the experience of the person who performs the test is fundamental. The male patient explicit his doubts and concerns in interpreting the meaning of the term “manually.”

1 D So this is the only test that I suggest you do here or in “X” [name of another health care facility]

*Questo ecco è l’unico esame che le consiglio di fare qui::(.) o in alternativa a “X” [nome di un’altra struttura sanitaria]*

2 MP uh

*ah*

3 D not elsewhere because it’s a test one of few tests that is still done manually

*diciamo non da altre parti perché è un esame è uno dei pochissimi esami che ancora viene eseguito manualmente*

4 MP uh huh

*mh mh*

5 D so the lab technician that looks at it and their experience is important

*quindi l’operatore che lo vede e la sua esperienza è fondamentale*

6 MP uh huh

*mh mh*

7 D since it’s not a simple, pleasant test

*dato che non è un esamino simpaticissimo*

**8 MP um I don’t think I understood what do you mean manually?**

***cioè non ho capito manualmente cosa vuol dire?***

The patient explicitly stated that he was not able to understand what “manually” meant or that the interpretation he achieved was not acceptable. This misunderstanding might have been also facilitated by the combination of technical (e.g., “manually”) and common (ambiguous) language (e.g., “pleasant test”). Overall, this example shows how both informative complexity and emotional concerns due to the intimate topics touched can explain (actual or potential) difficulties in understanding, and especially lack of understandings and semantic alternative understandings.

## Discussion

The present study systematically analyzed the main communication problems affecting the co-construction of shared understanding in first and follow-up ART triadic visits. It shed light on the different types of misunderstandings in these consultations, thus providing indications on which types of misunderstandings most frequently occur, when, by whom, and (in case of strong misunderstandings) about what. This is a never-explored area in the ART field: doctor-patient communication, in general, has been poorly investigated in the ART field, and misunderstandings have rarely been explored in medical fields.

Findings from this study showed that misunderstandings with acceptable evidence (clarifications, checks for understanding) are the most represented in ART triadic consultations. This finding confirms results obtained in other medical settings: in another study using the same coding scheme on a corpus of consultations with patients affected by diabetes, “acceptable evidence” of misunderstandings were again the most represented (Rossi and Macagno, 2020). These findings are coherent also with what has been suggested in the field of applied linguistics: overt corrections are in general not predominant in human interactions, also beyond the medical setting (e.g., Schegloff et al., 1977; Healey and Thirlwell, 2002; Kitzinger, 2012; Dingemanse et al., 2015, 2016). The prevalence of misunderstandings with acceptable evidence in first visits can be seen as an indication of the complexity of these ART interactions, confirming that first visits are usually longer and with more complex information exchanges than follow-up visits (Fossum and Arborelius, 2004; Graugaard et al., 2005). Such complexity, at a time when the relationship between couples and doctors still needs to be established, makes the efforts of reaching a shared understanding frequent and potentially challenging. Both clarifications and checks for understanding dropped drastically in the follow-up visits performed by the same doctors. For patients, however, numbers revealed a different trend: the same clarifications and checks slightly increased in follow-up visits, showing that efforts to build a shared understanding persist for patients in follow-ups.

The amount of semantic alternative understandings was remarkable in ART consultations and almost doubled those found in consultations with patients affected by type 2 diabetes using the same coding scheme (Rossi and Macagno, 2020), weighting for the number of turns. Indeed, in diabetic consultations, the pragmatic dimension was much more problematic than the semantic, suggesting diversity in clinical purposes more oriented toward self-management and lifestyle change that can more easily lead to wrong inferences about what the doctor says. Such prevalence of semantic alternative understandings in ART visits, at the opposite, may emphasize dialogic mismatches at the level of the specific semantic representation or content of what is said: alternative interpretations due to ambiguities, meanings interpreted too narrowly or broadly, or mistakes in identifying proper references are all phenomena falling in the semantic alternative understanding category. As shown by the example discussed in the previous section, this may indicate a greater complexity of the ART field at the level of the information and technical terms conveyed, also considering the complexity of treatments and procedures. We have found several examples in which a mixed-use of technical language and jargon language may have introduced ambiguity and caused problems of understanding, similarly to what was observed in the context of diabetes (Macagno and Rossi, 2019, 2021).

Concerning our findings related to who expressed the misunderstanding, we found that doctors may not completely understand patients’ statements, especially in first visits. Previous studies have shown that first visits have extensive and quite complex segments of history-taking (Fossum and Arborelius, 2004; Graugaard et al., 2005), which can explain the high numbers of misunderstandings contained in doctors’ turns. Within the couple, even if male patients participated overall less to the dialogue in terms of turns uttered compared to female patients, we found that they expressed a higher number of misunderstandings than female patients weighting for their number of turns. This may indicate that the quality of the contribution of male patients is high (i.e., they do not fear to express, potentially or problematic, alternative understandings, thus contributing to the shared effort of doctors and patients of resolving ambiguities and building a common understanding), even if they talk less. In the literature, it is known that male patients talk less than female patients in ART interactions (Leone et al., 2018; Borghi et al., 2019). The finding on male misunderstandings may shed light on the role of male patients in the ART consultation and care process. From the analysis of topics, we have also revealed how bureaucratic topics are frequently addressed by male patients, often introducing juridical complexity into the conversation. Male patients may provide important contributions to disambiguate specific contents of the medical visits that are relevant for the care process and that, if unclear, may result in dissatisfaction with ART care. Such specific conversational behavior expressed by male patients may be interpreted in the context of the social role theory (Eagly et al., 2000), with bureaucratic issues handled more often by male patients as a social role expectation to fulfill a family function more related to the practicalities of the daily life. However, their willingness to contribute especially when bureaucratic topics are discussed might also be interpreted as a way to convey their distress and anxiety in a more indirect way, by discussing less emotional topics. Our results may indirectly confirm findings about gender differences reported in previous studies, with male patients expressing in general less emotional reactions and psychological distress than female patients (Jordan and Revenson, 1999; Peterson et al., 2006; Nagórska et al., 2019). Further studies are needed to deepen and explore in other contexts the specific role of male patients in triadic consultations.

The analysis of topics of clearly expressed misunderstandings with strong evidence revealed the main contents of the consultations that may need specific attention: the long history-taking of the first visits can particularly generate ambiguities that doctors may need to explicit, and treatment- and consultation-related misunderstandings may particularly raise in follow-up visits by couples. Other topics that can generate difficulties in comprehension in ART interaction are bureaucratic, emotional, and relationship-building topics, revealing the unclear rules and administrations that feature the ART care together with emotionally and relationally charged aspects (probably due to the intimate aspects touched by the ART care). This overview of challenging topics may provide indications for ART doctors about contents that need particular clarity and attention to avoid ambiguities, and that can be also easily recognized and solved compared to more subtle types of misunderstandings.

## Limitations

This study has some limitations. The sample of visits was collected in 2013–2015, thus reflecting ART care and regulations of that period. Regulations in Italy have changed after that period, and this may have changed some of the topics of the conversation and the related possibility of misunderstandings. This mostly concerns misunderstandings about bureaucratic topics. The same can apply to ART visits in Countries with other regulations.

Concerning the use of the coding scheme, Cohen’s Kappa values were low for the two weak categories of no uptake and irrelevance. While these categories may need to be revised to fit the ART field or better defined, the low scores may depend on the fact that Cohen’s Kappa values are sensitive to the low numbers of occurrences.

Then, we did not analyze if and how problems of understanding were solved within the interaction and repaired. In this sense, this study should be complemented by a further study assessing the use of different types of repair operations and repair strategies (Schegloff, 1988; Healey and Thirlwell, 2002; Healey et al., 2005; Dingemanse et al., 2015; Albert and de Ruiter, 2018).

Finally, we have analyzed only misunderstandings as defined by Rossi and Macagno (2020). Other types of mismatches have been excluded by our analysis, like misconceptions and disagreements, which we, however, observed in the corpus. Further studies will extend the analysis of misunderstandings to other types of communication mismatches.

## Conclusion

This study showed that understanding can be problematic in ART triadic interactions. Such difficulty involved mostly the history-taking part of first visits, with doctors’ requests for clarification and checks, and the treatment-related and medical consultations topics in follow-up visits, with couples’ direct expressions of misunderstanding. It also highlighted the role of male patients in contributing to expressing specific problems of understanding. Compared to other consultations, ART visits may be particularly rich in information exchanges and technicalities together with complex regulations and intimate, emotionally charged contents, thus explaining the different types of misunderstandings observed. ART doctors should be aware of this complexity, and try to disambiguate as much as possible terms and concepts in specific phases and topics of the consultation, as well as be sensitive to couples’ signs of problematic understanding, which are usually direct and clear.

## Data Availability Statement

The data analyzed in this study is subject to the following licenses/restrictions: the datasets analyzed during the current study are available from the corresponding author on reasonable request. Requests to access these datasets should be directed to MGR, mgrazia.rossi@fcsh.unl.pt.

## Ethics Statement

The research project was approved by the Ethical Review Board of the University of Milan and by the Ethical Review Boards of the eight participating ART clinics. Written informed consent was obtained from each participant included in the study, and patients and physicians were guaranteed the right to withdraw voluntarily if they so decide. Data were managed according to local regulations regarding privacy.

## Author Contributions

All the authors contributed to the conception and design of the work, revised it critically, and gave their final approval of the version to be published. EV contributed to the acquisition of data. MGR and JM contributed to the data analysis and the draft of the work.

## Conflict of Interest

The authors declare that the research was conducted in the absence of any commercial or financial relationships that could be construed as a potential conflict of interest.

## Appendix

**TABLE A1:** Topics and subtopics used to classify strong types of misunderstandings.

Topics and sub-topics	
**(A) Bureaucratic topics: Country and center rules**	
**Country rules**	• Regulations in egg donation and heterologous insemination (Italy) • Regulations *in vitro* fertilization (Italy) • Identification of a foreign country allowing egg donation
**Center rules**	• Coordination • Treatment costs • Contact methods
**B. Treatment topics: Timing and procedures**	
**Timing**	• Examinations (spermiogram, blood test, fallopian tubes) • Waiting list duration • Medical visits
**Procedures**	• After getting pregnant • Treatments (sperm freezing, embryo transfer, stimulation) • Assessment of treatment options (egg and/or sperm donation) • In a foreign medical center • Informed consent compilation • Monitoring (number of patients, number of visits before embryo transfer or stimulation cycles of insemination)
**(C) Clinical consultation topics: Medical information**	
	• Assessment of current medical condition • Results of the fallopian tubes examination • Issues related to ovodonation • Issues related to ovulation and menstrual cycle • Issues related to embryo transfer • Issues related to (natural) insemination • Issues related to intrauterine pregnancy • Issues related to heterologous fecundation • Issues related to examinations • Fertility rate • Terminological issues (embryo freezing, karyotype, antagonist stimulation, follow up)
**(D) History-taking topics**	
	• Biographical information (location of couple’s provenience, marital status, patients’ age) • Familiar anamnestic information (parents’ menopause threshold) • Patients’ anamnestic information (menstrual cramps and/or menstrual cycle, previous pregnancies, male sexual problems, sexual intercourse, other health issues) • Previous examinations (sperm test, blood test, breast ultrasound examination) • Relevant documentation • Drug-taking (identification or dosage) • Previous access to a different medical center (discussion of previous treatment place, discussion of clinical issues in another center) • Previous treatment (*in vitro* fertilization, interruption of a treatment)
**(E) Emotional topics: Concerns and complaints**	
**Concerns**	• Ovulation and menstrual cycle • Preventive measures related to embryo transfer or stimulation cycles of insemination • Stress caused by the treatment process • Complications of heterologous fecundation (due to fibromas) • Medical limitations
**Complaints**	• Financial values in another medical center • Treatment in another medical center (overtreatment, embryo procedure, sperm freezing)
**(F) Rapport-building topics**	
	• Previous contact and presentation • Mutual acquaintances • Informal comments and jokes

## References

[B1] Albert S. de Ruiter J. P. (2018). Repair: the Interface Between Interaction and Cognition. *Top. Cogn. Sci.* 10 279–313. 10.1111/tops.12339 29749039 PMC6849777

[B2] Angelelli C. (2004). *Medical Interpreting and Cross-cultural Communication.* Cambridge: Cambridge University Press.

[B3] Babore A. Stuppia L. Trumello C. Candelori C. Antonucci I. (2017). Male factor infertility and lack of openness about infertility as risk factors for depressive symptoms in males undergoing assisted reproductive technology treatment in Italy. *Fertil. Steril.* 107 1041–1047. 10.1016/j.fertnstert.2016.12.031 28202187

[B4] Bazzanella C. Damiano R. (1999a). “Coherence and Misunderstanding in Everyday Conversations,” in *Coherence in Spoken and Written Discourse: How to Create it and How to Describe it*, eds Bublitz W. Lenk U. Ventola E. (Amsterdam: John Benjamins Publishing Company), 175–187. 10.1075/pbns.63.13baz

[B5] Bazzanella C. Damiano R. (1999b). The interactional handling of misunderstanding in everyday conversations. *J. Pragmat.* 31 817–836. 10.1016/S0378-2166(98)00058-7

[B6] Berger Z. D. Boss E. F. Beach M. C. (2017). Communication behaviors and patient autonomy in hospital care: a qualitative study. *Patient Educ. Couns.* 100 1473–1481. 10.1016/j.pec.2017.03.006 28302341

[B7] Borghi L. Leone D. Poli S. Becattini C. Chelo E. Costa M. (2019). Patient-centered communication, patient satisfaction, and retention in care in assisted reproductive technology visits. *J. Assist. Reprod. Genet.* 36 1135–1142. 10.1007/s10815-019-01466-1 31077010 PMC6603100

[B8] Braun V. Clarke V. (2006). Using thematic analysis in psychology. *Qual. Res. Psychol.* 3 77–101. 10.1191/1478088706qp063oa 32100154

[B9] Chandra S. Mohammadnezhad M. Ward P. (2018). Trust and Communication in a Doctor- Patient Relationship: a Literature Review. *J. Healthc. Commun.* 03 1–6. 10.4172/2472-1654.100146

[B10] Courbiere B. Lacan A. Grynberg M. Grelat A. Rio V. Arbo E. (2020). Psychosocial and professional burden of Medically Assisted Reproduction (MAR): results from a French survey. *PLoS One* 15:e0238945. 10.1371/journal.pone.0238945 32970695 PMC7514013

[B11] Cox A. (2017). *The Dynamics of (mis)Communication in Language Discordant Multi-Party Consultations in the Emergency Department.* Ph. D. thesis. Belgium: Katholieke Universiteit Leuven. Available online at: https://lirias.kuleuven.be/handle/123456789/640782.

[B12] Cox A. Li S. (2020). The medical consultation through the lenses of language and social interaction theory. *Adv. Heal. Sci. Educ.* 25 241–257. 10.1007/s10459-018-09873-2 30715620 PMC7018671

[B13] Crawford T. Candlin S. Roger P. (2017). New perspectives on understanding cultural diversity in nurse–patient communication. *Collegian* 24 63–69. 10.1016/j.colegn.2015.09.001 29218964

[B14] Di Blasi Z. Harkness E. Ernst E. Georgiou A. Kleijnen J. (2001). Influence of context effects on health outcomes: a systematic review. *Lancet* 357 757–762. 10.1016/s0140-6736(00)04169-6 11253970

[B15] Dingemanse M. Kendrick K. H. Enfield N. J. (2016). A coding scheme for other-initiated repair across languages. *Open Linguist.* 2 35–46. 10.1515/opli-2016-0002

[B16] Dingemanse M. Roberts S. G. Baranova J. Blythe J. Drew P. Floyd S. (2015). Universal principles in the repair of communication problems. *PLoS One* 10:e0136100. 10.1371/journal.pone.0136100 26375483 PMC4573759

[B17] Eagly A. H. Wood W. Diekman A. B. (2000). “Social role theory of sex differences and similarities: a current appraisal,” in *The Developmental Social Psychology of Gender*, eds Eckes T. Trautner H. M. (Mahwah: Erlbaum), 123–174.

[B18] Epstein R. M. Street R. L. J. (2011). Shared mind: communication, decision making, and autonomy in serious illness. *Ann. Fam. Med.* 9 454–461. 10.1370/afm.1301 21911765 PMC3185482

[B19] Fossum B. Arborelius E. (2004). Patient-centred communication: videotaped consultations. *Patient Educ. Couns.* 54 163–169. 10.1016/S0738-3991(03)00208-8 15288910

[B20] Gameiro S. Boivin J. Peronace L. Verhaak C. M. (2012). Why do patients discontinue fertility treatment? A systematic review of reasons and predictors of discontinuation in fertility treatment. *Hum. Reprod. Update* 18 652–669. 10.1093/humupd/dms031 22869759 PMC3461967

[B21] Graugaard P. K. Holgersen K. Eide H. Finset A. (2005). Changes in physician-patient communication from initial to return visits: a prospective study in a haematology outpatient clinic. *Patient Educ. Couns.* 57 22–29. 10.1016/j.pec.2004.03.014 15797149

[B22] Greil A. L. (1997). Infertility and psychological distress: a critical review of the literature. *Soc. Sci. Med.* 45 1679–1704. 10.1016/s0277-9536(97)00102-0 9428088

[B23] Gulbrandsen P. Lindstrøm J. C. Finset A. Hall J. A. (2020). Patient affect, physician liking for the patient, physician behavior, and patient reported outcomes: a modeling approach. *Patient Educ. Couns.* 103 1143–1149. 10.1016/j.pec.2020.01.003 31964578

[B24] Hagihara A. Tarumi K. Odamaki M. Nobutomo K. (2005). A signal detection approach to patient-doctor communication and doctor-shopping behaviour among Japanese patients. *J. Eval. Clin. Pract.* 11 556–567. 10.1111/j.1365-2753.2005.00581.x 16364109

[B25] Hall J. A. Roter D. L. Katz N. R. (1988). Meta-analysis of correlates of provider behavior in medical encounters. *Med. Care* 26 657–675. 10.1097/00005650-198807000-00002 3292851

[B26] Healey P. G. T. Colman M. Thirlwell M. (2005). “Analysing Multimodal Communication,” in *Advances in Natural Multimodal Dialogue Systems*, eds van Kuppevelt J. C. J. Dybkjær L. Bernsen N. O. (Dordrecht: Springer Netherlands), 113–129. 10.1007/1-4020-3933-6_6

[B27] Healey P. G. T. Thirlwell M. (2002). “Analysing Multi-Modal Communication: repair-Based Measures of Communicative Co-ordination,” in *Proceedings of International CLASS Workshop on Natrual, Intelligent and Effective Interaction in Multimodal Dialogue Systems*, eds Kuppevelt J. V. Dybkjær L. Bernsen N. O. (Denmark: University of Southern Denmark), 83–92.

[B28] Jefferson G. (2004). “Glossary of transcript symbols with an introduction,” in *Conversation Analysis: Studies from the First Generation*, ed. Lerner G. H. (Amsterdam: John Benjamins), 13–31. 10.1075/pbns.125.02jef

[B29] Jordan C. Revenson T. A. (1999). Gender differences in coping with infertility: a meta-analysis. *J. Behav. Med.* 22 341–358. 10.1023/A:1018774019232 10495967

[B30] Kitzinger C. (2012). “Repair,” in *The Handbook of Conversation Analysis*, eds Sidnell J. Stivers T. (Oxford: John Wiley & Sons), 229–256.

[B31] Leone D. Borghi L. Del Negro S. Becattini C. Chelo E. Costa M. (2018). Doctor–couple communication during assisted reproductive technology visits. *Hum. Reprod.* 33 877–886. 10.1093/humrep/dey069 29635461

[B32] Leone D. Menichetti J. Barusi L. Chelo E. Costa M. De Lauretis L. (2017). Breaking bad news in assisted reproductive technology: a proposal for guidelines. *Reprod. Health* 14:87. 10.1186/s12978-017-0350-1 PMC5520370 28728610

[B33] Levinson W. Roter D. L. Mullooly J. P. Dull V. T. Frankel R. M. (1997). Physician-patient communication. The relationship with malpractice claims among primary care physicians and surgeons. *JAMA* 277 553–559. 10.1001/jama.277.7.553 9032162

[B34] Lynch D. J. McGrady A. V. Nagel R. W. Wahl E. F. (2007). The patient-physician relationship and medical utilization. *Prim. Care Companion J. Clin. Psychiatry* 9 266–270. 10.4088/pcc.v09n0403 17934550 PMC2018838

[B35] Macagno F. Rossi M. G. (2019). Metaphors and problematic understanding in chronic care communication. *J. Pragmat.* 151 103–117. 10.1016/j.pragma.2019.03.010

[B36] Macagno F. Rossi M. G. (2021). “The communicative functions of metaphors between explanation and persuasion,” in *Inquiries in philosophical pragmatics-Theoretical developments*, eds Macagno F. Capone A. (Switzerland: Springer), 171–191. 10.1007/978-3-030-56437-7_12

[B37] McCabe R. Healey P. G. T. (2018). Miscommunication in Doctor–Patient Communication. *Top. Cogn. Sci.* 10 409–424. 10.1111/tops.12337 29749042 PMC6033118

[B38] McHugh M. L. (2012). Lessons in biostatistics interrater reliability: the kappa statistic. *Biochem. Med.* 22 276–282. Available online at: https://hrcak.srce.hr/89395 PMC3900052 23092060

[B39] Nagórska M. Bartosiewicz A. Obrzut B. Darmochwał-Kolarz D. (2019). Gender differences in the experience of infertility concerning polish couples: preliminary research. *Int. J. Environ. Res. Public Health* 16:2337. 10.3390/ijerph16132337 31269703 PMC6651646

[B40] Paternotte E. van Dulmen S. van der Lee N. Scherpbier A. J. J. A. Scheele F. (2015). Factors influencing intercultural doctor–patient communication: a realist review. *Patient Educ. Couns.* 98 420–445. 10.1016/j.pec.2014.11.018 25535014

[B41] Peterson B. D. Newton C. R. Rosen K. H. Skaggs G. E. (2006). Gender differences in how men and women who are referred for IVF cope with infertility stress. *Hum. Reprod.* 21 2443–2449. 10.1093/humrep/del145 16675482

[B42] Purewal S. Chapman S. C. E. van den Akker O. B. A. (2017). A systematic review and meta-analysis of psychological predictors of successful assisted reproductive technologies. *BMC Res. Notes* 10:711. 10.1186/s13104-017-3049-z 29212545 PMC5719749

[B43] Roberts C. Moss B. Wass V. Sarangi S. Jones R. (2005). Misunderstandings: a qualitative study of primary care consultations in multilingual settings, and educational implications. *Med. Educ.* 39 465–475. 10.1111/j.1365-2929.2005.02121.x 15842680

[B44] Roberts C. Sarangi S. (2005). Theme oriented discourse analysis of medical encounters. *Med. Educ.* 39 632–640. 10.1111/j.1365-2929.2005.02171.x 15910440

[B45] Rossi M. G. Leone D. Bigi S. (2017). The ethical convenience of non-neutrality in medical encounters: argumentative instruments for healthcare providers. *Teoria* 37 139–157. 10.4324/9780429453441-20

[B46] Rossi M. G. Macagno F. (2020). Coding Problematic Understanding in Patient–provider Interactions. *Health Commun.* 35 1487–1496. 10.1080/10410236.2019.1652384 31460797

[B47] Rossi M. G. Macagno F. (2021). “Intercultural pragmatics in healthcare communication: an overview of the field,” in *Cambridge Handbook of Intercultural Pragmatics*, ed. Kecskes I. (Cambridge: Cambridge University Press).

[B48] Samani R. O. Vesali S. Navid B. Vakiliniya B. Mohammadi M. (2017). Evaluation on hope and psychological symptoms in infertile couples undergoing assisted reproduction treatment. *Int. J. Fertil. Steril.* 11 123–129. 28670431 10.22074/ijfs.2017.4838 PMC5347450

[B49] Schegloff E. (1988). Presequences and indirection: applying speech act theory to ordinary conversation. *J. Pragmat.* 12 55–62. 10.1016/0378-2166(88)90019-7

[B50] Schegloff E. Jefferson G. Sacks H. (1977). The preference for self-correction in the organization of repair in conversation. *Language* 53 361–382. 10.2307/413107

[B51] Schouten B. Meeuwesen L. (2006). Cultural differences in medical communication: a review of the literature. *Patient Educ. Couns.* 64 21–34. 10.1016/j.pec.2005.11.014 16427760

[B52] Stanhiser J. Steiner A. Z. (2018). Psychosocial aspects of fertility and assisted reproductive technology. *Obstet. Gynecol. Clin.* 45 563–574. 10.1016/j.ogc.2018.04.006 30092929

[B53] Stewart M. A. (1995). Effective physician-patient communication and health outcomes: a review. *CMAJ* 152 1423–1433. 7728691 PMC1337906

[B54] Street R. L. J. (2013). How clinician-patient communication contributes to health improvement: modeling pathways from talk to outcome. *Patient Educ. Couns.* 92 286–291. 10.1016/j.pec.2013.05.004 23746769

[B55] Street R. L. J. Makoul G. Arora N. Epstein R. Street R. Jr. Makoul G. (2009). How does communication heal? Pathways linking clinician–patient communication to health outcomes. *Patient Educ. Couns.* 74 295–301. 10.1016/j.pec.2008.11.015 19150199

[B56] Tzanne A. (2000). *Talking at Cross-purposes: The Dynamics of Miscommunication.* Amsterdam: John Benjamins Publishing.

[B57] Van den Broeck U. D’Hooghe T. Enzlin P. Demyttenaere K. (2010). Predictors of psychological distress in patients starting IVF treatment: infertility-specific versus general psychological characteristics. *Hum. Reprod.* 25 1471–1480. 10.1093/humrep/deq030 20338959

[B58] Van den Broeck U. Holvoet L. Enzlin P. Bakelants E. Demyttenaere K. D’Hooghe T. (2009). Reasons for dropout in infertility treatment. *Gynecol. Obstet. Invest.* 68 58–64. 10.1159/000214839 19401627

[B59] Van Dulmen A. M. Verhaak P. F. M. Bilo H. J. G. (1997). Shifts in doctor-patient communication during a series of outpatient consultations in non-insulin-dependent diabetes mellitus. *Patient Educ. Couns.* 30 227–237. 10.1016/S0738-3991(96)00965-2 9104379

[B60] Verdonik D. (2010). Between understanding and misunderstanding. *J. Pragmat.* 42 1364–1379. 10.1016/j.pragma.2009.09.007

[B61] Weigand E. (1999). Misunderstanding: the standard case. *J. Pragmat.* 31 763–785. 10.1016/s0378-2166(98)00068-x

[B62] Yus F. (1999). Misunderstandings and explicit/implicit communication. *Pragmatics* 9 487–517. 10.1075/prag.9.4.01yus 33486653

[B63] Zolnierek K. B. H. Dimatteo M. R. (2009). Physician communication and patient adherence to treatment: a meta-analysis. *Med. Care* 47 826–834. 10.1097/MLR.0b013e31819a5acc 19584762 PMC2728700

